# Effect of pore diameter on the elution behavior of analytes from thermoresponsive polymer grafted beads packed columns

**DOI:** 10.1038/s41598-021-89165-9

**Published:** 2021-05-11

**Authors:** Kenichi Nagase, Yuta Umemoto, Hideko Kanazawa

**Affiliations:** Faculty of Pharmacy, Keio University, 1-5-30 Shibakoen, Minato, Tokyo, 105-8512 Japan

**Keywords:** Analytical chemistry, Green chemistry

## Abstract

Temperature-responsive chromatography using thermoresponsive polymers is innovative and can control analyte retention via column temperature. Analyte elution behavior in this type of chromatography depends on the modified thermoresponsive polymer and the structure of the base materials. In the present study, we examine the effect of the pore diameter of silica beads on analyte elution behavior in temperature-responsive chromatography. Poly(*N*-isopropylacrylamide-*co*-*n*-butyl methacrylate) hydrogel was applied to beads of various pore sizes: 7, 12, and 30 nm. Almost the same amount of copolymer hydrogel was applied to all beads, indicating that the efficiency of copolymer modification was independent of pore size. Analyte retention on prepared beads in a packed column was observed using steroids, benzodiazepines, and barbiturates as analytes. Analyte retention times increased with temperature on packed columns of 12- and 30-nm beads, whereas the column packed with 7-nm beads exhibited decreased retention times with increasing temperature. The difference in analyte elution behavior among the various pore sizes was attributed to analyte diffusion into the bead pores. These results demonstrate that bead pore diameter determines temperature-dependent elution behavior.

## Introduction

Liquid chromatography is one of the most effective separation analysis methods across several biomedical fields^1–3^. A variety of stationary phases for liquid chromatography have been developed for various applications. One of them is using thermoresponsive polymer, poly(*N*-isopropylacrylamide) (PNIPAAm), modified stationary phase for temperature-responsive chromatography^4–7^. PNIPAAm exhibits thermoresponsive hydrophilic and hydrophobic property changes that can be attributed to hydration and dehydration in response to temperature change^8,9^. Since the phase transition temperature of PNIPAAm (32 °C) is close to the human body temperature, it has relevance to various biomedical applications such as drug and gene delivery systems^10–14^, biosensors and bioimaging systems^15–19^, temperature-modulated cell separation systems^20–23^, and cell culture substrates for tissue engineering^24–27^. In a chromatography system, PNIPAAm-modified silica beads are used as the stationary phase, and hydrophobic interaction between PNIPAAm and the analyte is modulated by changing temperature (via temperature-responsive PNIPAAm hydrophobicity changes). The chromatography system uses all-aqueous mobile phases, although the addition of a small amount of methanol is required to improve detection with mass spectroscopy^28^. To improve the performance of chromatography systems, various types of PNIPAAm copolymer have been investigated such as the copolymerization of hydrophobic monomers to enhance hydrophobic interaction^29–32^ and the copolymerization of ionic monomers to utilize electrostatic interaction besides hydrophobic interaction^33–37^. Moreover, several types of PNIPAAm configuration on silica bead surfaces have been investigated using various polymerization methods^4,38–40^. Therefore, almost all studies on temperature-responsive chromatography focus the PNIPAAm on silica beads surface. However, although the structure of silica beads, especially pore diameter, is an essential factor in the separation efficiency of a chromatographic stationary phase, this has not been studied in temperature-responsive chromatography.

**Figure 1:**
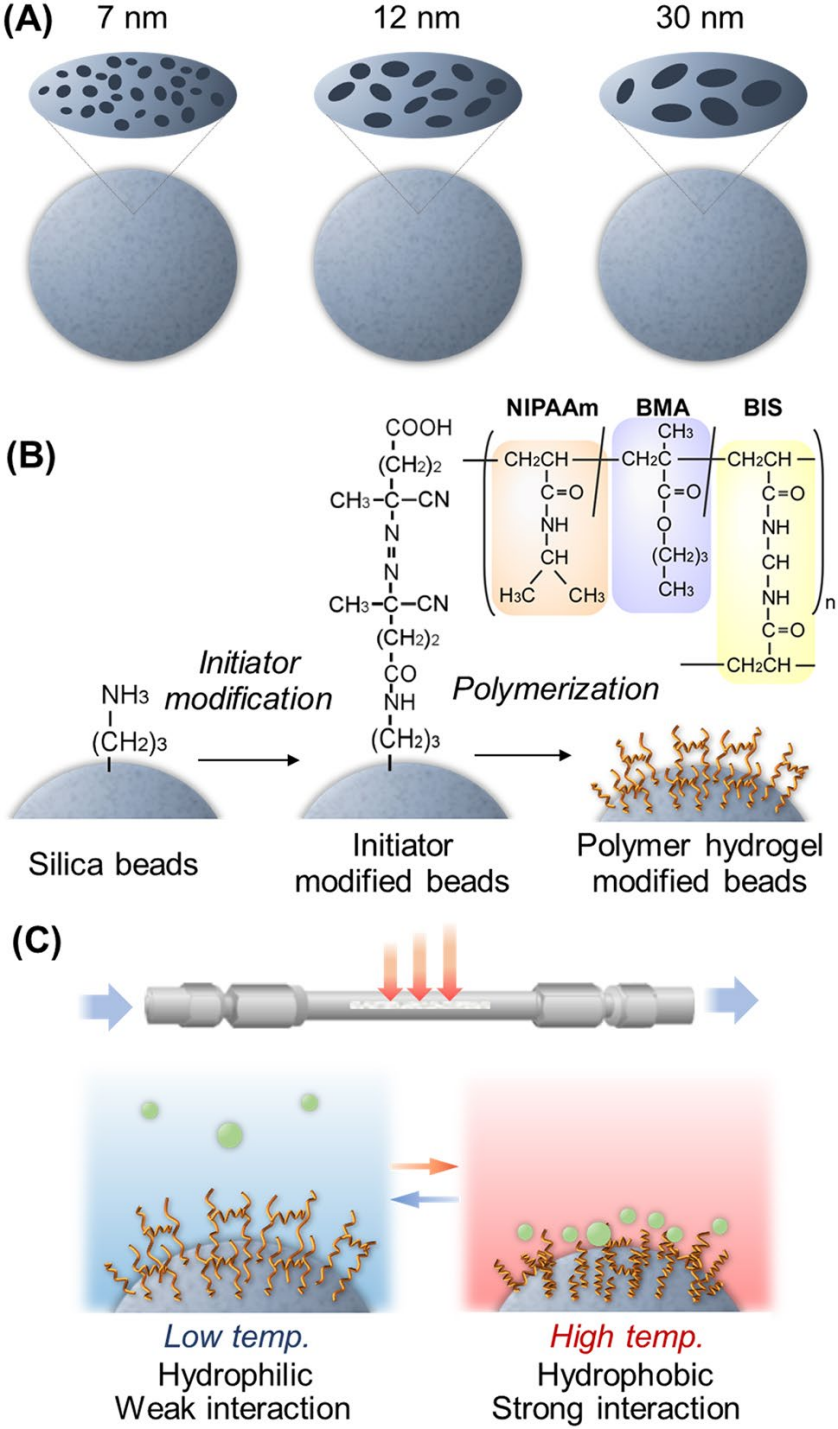
Schematic illustration of the preparation of thermoresponsive copolymer hydrogel-modified beads with various pores. (**A**) Porous silica beads with various diameters, (**B**) reaction scheme for the preparation of a hydrogel-modified column, and (**C**) temperature-modulated interaction with an analyte in an all-aqueous chromatography system.

In the present study, to investigate the effect of pore diameter on analyte elution in temperature-responsive chromatography, stationary phases were prepared using silica beads with various pore diameters (7, 12, and 30 nm) as base materials (Fig. 1). P(NIPAAm-*co*-*n*-butyl methacrylate (BMA)), a PNIPAAm copolymer with a hydrophobic co-monomer, hydrogel was used to modify the silica bead surfaces. The elution behavior of the packed column of prepared beads was assessed using steroids, benzodiazepines, and barbiturates as model analytes because these molecules have different chemical structures and hydrophobicities for temperature-responsive chromatography. Moreover, these analytes represent different types of drugs: steroids are anti-inflammatory drugs, whereas benzodiazepines and barbiturates are psychoactive drugs. Thus, the separation of these analytes would exhibit the versatility of drug analysis using temperature-responsive chromatography. Analyte diffusion into the pores was investigated using pullulan with a range of molecular weights. The effect of the pore diameter of the temperature-responsive stationary phase on the elution behavior of the analytes has been investigated.

## Results and discussion

### Characterization of beads with various pore diameters

The morphology of the beads was observed by field emission scanning electron microscopy (FE-SEM) before and after modification by the P(NIPAAm-*co*-BMA) copolymer hydrogel (Supplementary Fig. S1). Almost the same bead morphology was observed before and after polymerization. This result indicates that the aggregation and deformation of the beads did not occur as a result of the copolymer hydrogel modification procedure.

The prepared beads were characterized by elemental analysis (Table 1). The carbon contents of all beads with various pore sizes were increased after V-501 immobilization (in comparison with the carbon content of aminopropyl silica beads). This result indicates that V-501 modification was successfully performed via a condensation reaction between the amino group of the beads and the carboxyl group of V-501. The carbon content also increased after the polymerization reaction, indicating that the P(NIPAAm-*co*-BMA) copolymer was successfully modified through this reaction. The estimated amount of grafted polymer was almost the same among all beads. This result indicates that the efficiency of hydrogel modification on the silica bead surfaces was independent of the pore diameter of the silica beads.

**Table 1:** Characterization of temperature-responsive hydrogel-modified beads using CHN elemental analysis.

Pore diameter (nm)	Beads	Carbon composition (%)	%C_(calcd)_	Immobilized initiator (μmol/m^2^)	Grafted polymer (mg/m^2^)
7	Aminopropyl silica beads	4.00			
	Initiator-modified beads	5.55	51.4	0.657	
	P(NIPAAm-*co*-BMA)-modified beads	9.83	63.8		0.440
12	Aminopropyl silica beads	2.84			
	Initiator-modified beads	7.91	51.4	1.55	
	P(NIPAAm-*co*-BMA)-modified beads	13.74	63.8		0.448
30	Aminopropyl silica beads	2.50			
	Initiator-modified beads	4.72	51.4	1.63	
	P(NIPAAm-*co*-BMA)-modified beads	6.88	63.8		0.370

Carbon composition was determined through CHN elemental analysis. %C (calcd) was calculated as the percentage of the molecular weight of carbon in the initiator and copolymer. The amounts of initiator and polymer on silica beads were estimated using the carbon composition.

To investigate the porous property of the beads before and after polymer hydrogel modification, the pore diameter, pore volume, and surface area of the beads were investigated by nitrogen adsorption and using the Brunauer–Emmett–Teller (BET) method (Table 2)^41,42^. Relatively larger surface area and pore volume were observed in the 12-nm pore-diameter aminopropyl silica beads compared to those of 7- and 30-nm pore diameter, indicating that the 12-nm diameter beads had greater porosity compared to the other beads. This was probably due to the production method for 12-nm aminopropyl silica beads being slightly different than that for other pore-diameter beads. Pore diameter, pore volume, and surface area slightly decreased after polymerization because the polymer hydrogel modified the inside of the bead pores. However, the results indicated that the porous structure remained in all beads after copolymer hydrogel modification, i.e., the bead pores did not become clogged.

**Table 2:** Porous properties of the beads using nitrogen adsorption before and after polymer modification.

Pore diameter (nm)	Beads	Surface area (m^2^/g)	Total pore volume (cm^3^/g)	Peak pore diameter (nm)
7	Aminopropyl silica beads	169	0.331	10.8
	P(NIPAAm-*co*-BMA)-modified beads	159	0.460	8.31
12	Aminopropyl silica beads	252	0.910	14.1
	P(NIPAAm-*co*-BMA)-modified beads	180	0.568	12.6
30	Aminopropyl silica beads	99.0	0.680	27.3
	P(NIPAAm-*co*-BMA)-modified beads	94.3	0.568	24.1

The values were calculated using the Brunauer–Emmett–Teller (BET) method.

### Elution behavior of analytes through a packed column of beads

The prepared polymer hydrogel-modified beads were packed into a stainless-steel column (4.6 mm internal diameter × 100 mm length). The elution behavior of various analytes from the bead-packed column was observed.

**Figure 2:**
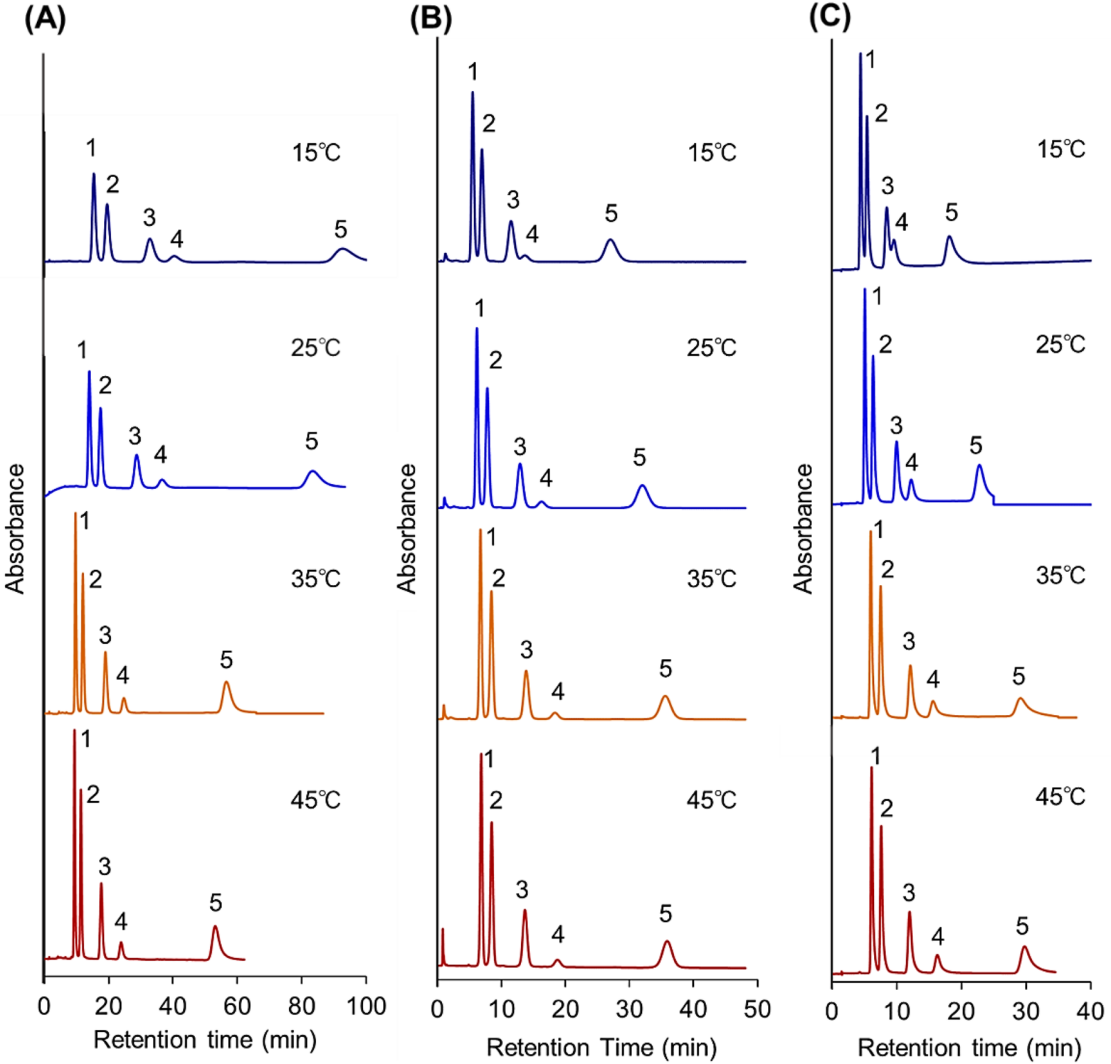
Chromatograms of steroids using a P(NIPAAm-*co*-BMA) hydrogel-modified bead column. (**A**) 7-nm pore-diameter beads, (**B**) 12-nm pore-diameter beads, and (**C**) 30-nm pore-diameter beads as base materials. The mobile phase was pure water, and the elution of the analytes was monitored at 254 nm at a mobile-phase flow rate of 1.0 min/L. Peak 1 = hydrocortisone, peak 2 = prednisolone, peak 3 = dexamethasone, peak 4 = hydrocortisone acetate, and peak 5 = testosterone.

Five hydrophobic steroids with similar molecular weights and various hydrophobicities were used as analytes (Table S1). The temperature-dependent elution behavior of the hydrophobic steroids was observed (Fig. 2). To confirm the replication of the analyte elution behavior, the steroid retention time measurements were repeated five times (Table S2 and Supplementary Fig. S2). Retention time scarcely changed over the repeated measurements, indicating that the prepared temperature-responsive column exhibited reproducible elution behavior.

On all columns, the retention time of the steroids increased with increasing analyte hydrophobicity, Log*P*. This result indicates that steroid retention occurred via hydrophobic interaction between the polymer and analytes. Packed columns of beads with 12- and 30-nm pore diameters exhibited an increase in the retention time of the hydrophobic steroids with increasing column temperature, and effective separation was observed at the highest temperature of 45 °C. For example, dexamethasone and hydrocortisone acetate were eluted as an overlapped peak at 15 °C. With increasing temperature, dexamethasone and hydrocortisone acetate were separated and eluted as two separate peaks. This is because the enhanced hydrophobic interaction between copolymer hydrogel on the silica beads and the hydrophobic steroids could be attributed to the dehydration of the copolymer on the bead surfaces at a higher temperature. By contrast, beads packed column with 7-nm pore-diameter beads exhibited decreasing analyte retention times with increasing column temperature. The differences in the retention profiles were likely attributable to the pore diameter of the base beads.

For all the columns, the peak widths decreased with increasing temperature. This is attributed to the temperature-dependent shrinkage of the thermoresponsive polymer hydrogel layer on the silica bead surface. At low temperatures, this polymer layer was swollen, and the analyte tended to diffuse into it. Thus, relatively broad peaks were observed at low column temperatures. Contrastingly, the silica bead polymer layers shrunk at high temperatures, and the analyte did not tend to diffuse into the hydrogel layer, leading to relatively sharp peaks at high temperatures.

By comparing peak shapes among the various pore diameter sizes at high temperatures, sharp peaks were observed for the small pore-diameter-bead packed column compared to the large pore-diameter-bead packed column. This is probably attributable to the difference in the tendency of the analyte to diffuse into the pores. For large pore-diameter beads, the analyte tended to diffuse into the pores and interact with the polymer hydrogel layer inside the silica beads, leading to broad peaks.

**Figure 3:**
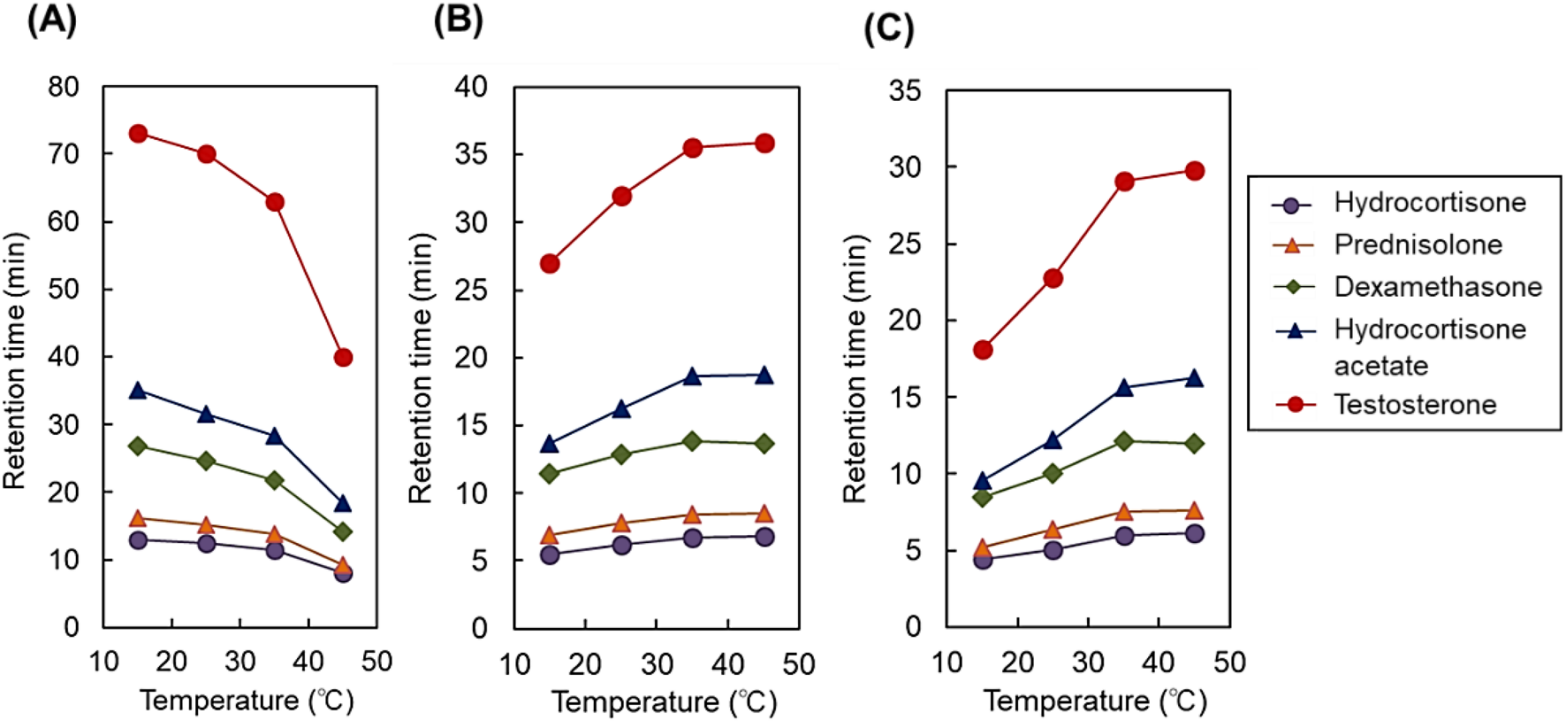
Retention time of hydrophobic steroids using P(NIPAAm-*co*-BMA) hydrogel-modified bead columns with (**A**) 7-nm pore-diameter beads, (**B**) 12-nm pore-diameter beads, and (**C**) 30-nm pore-diameter beads as base materials.

To investigate the relationship between retention time and column temperature, changes in the retention time profile with temperature and Van’t Hoff plots for the steroids were obtained (Fig. 3 and Supplementary Fig. S3, respectively). With the 12- and 30-nm pore-diameter beads, retention time increased with column temperature, showing the same tendency as previously reported temperature-responsive chromatography columns (Fig. 3B,C and Supplementary Fig. S3B,C)^4,43,44^. Large increases in the retention time of steroids were observed in the lower temperature regions. This is because of the phase transition temperature of the copolymer used on the beads. A similar P(NIPAAm-*co*-BMA) copolymer hydrogel exhibited phase transition at approximately 20 °C^21^. Thus, modified hydrogel dehydration likely occurred in this temperature region, leading to the retention of the steroids through enhanced hydrophobic interaction. By contrast, on the 7-nm pore-diameter beads, retention time decreased with increasing column temperature (Fig. 3A and Supplementary Fig. S3A).

To investigate the effect of pore diameter on the steroid separation performance, the separation factors between hydrocortisone and testosterone were observed (Supplementary Fig. S4). A large increase in separation factor with increasing temperature was observed in the large 30-nm pore-diameter column, and a modest increase was observed in the 12-nm pore-diameter beads. In contrast, for the 7-nm pore-diameter beads, a decrease in separation factor was observed with increasing temperature. This result was similar to the tendency of the change in retention times in the columns. Thus, the separation performance of the beads could be related to the different bead retention profiles.

These results demonstrate that a column using small pore-diameter beads (7 nm) can control analyte retention via changes in column temperature in a manner opposite to that previously reported for temperature-responsive chromatography columns^4,43,44^. The small pore-diameter-bead packed column achieved high-resolution separation at low temperatures, whereas the relatively large pore-diameter-bead packed columns (12 and 30 nm) exhibited low-resolution separation at low temperatures. Thus, the developed small pore-diameter beads are useful for the separation of an analyte at low temperatures, which is advantageous for the separation of analytes that tend to deactivate and decompose at relatively high temperatures.

**Figure 4:**
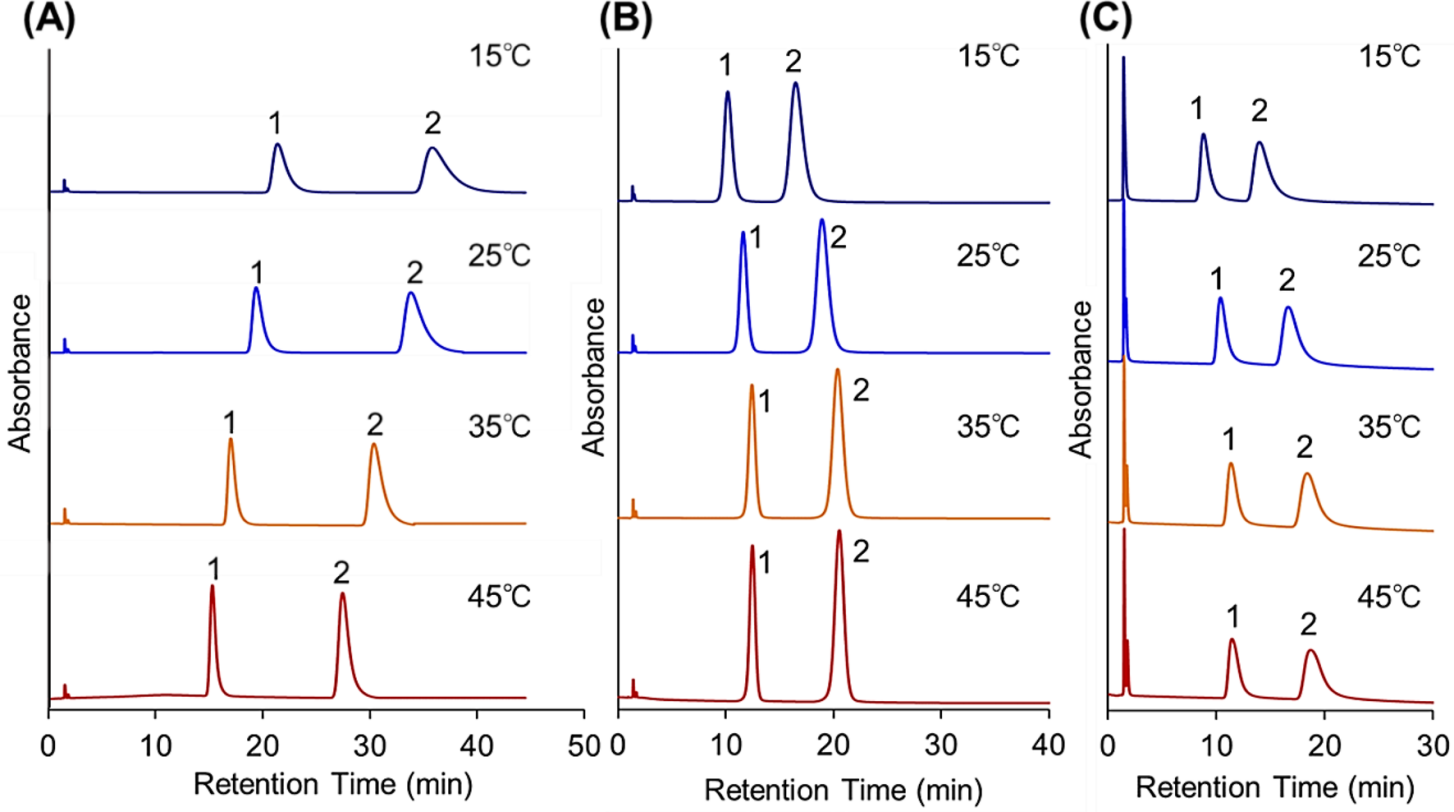
Chromatograms of benzodiazepines using P(NIPAAm-*co*-BMA) hydrogel-modified bead columns with (**A**) 7-nm pore-diameter beads, (**B**) 12-nm pore-diameter beads, and (**C**) 30-nm pore-diameter beads as base materials. Mobile phase was pure water. Elution of analytes was monitored at 220 nm at a mobile-phase flow rate of 1.0 min/L. Peak 1 = flunitrazepam; peak 2 = diazepam.

**Figure 5:**
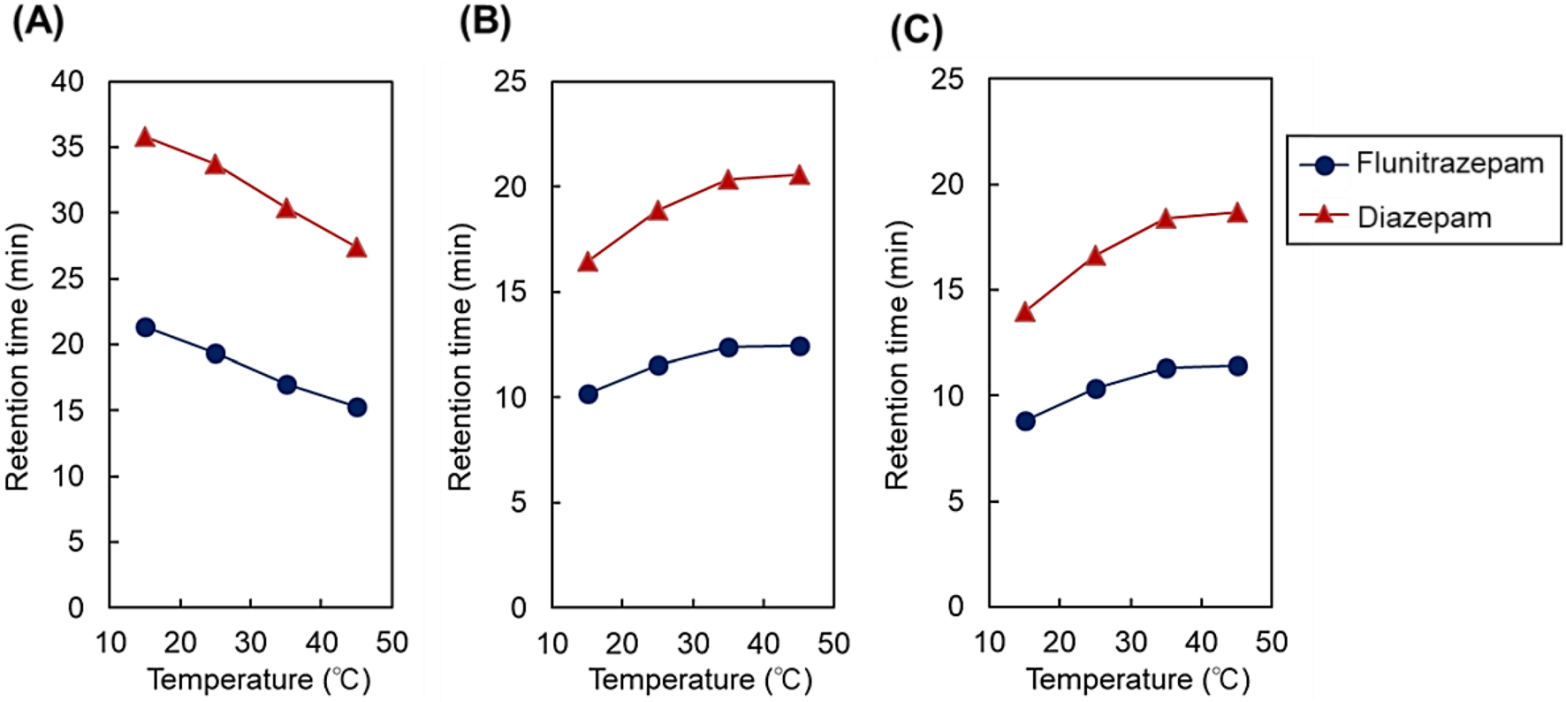
Retention time of benzodiazepines using P(NIPAAm-*co*-BMA) hydrogel-modified bead columns with (**A**) 7-nm pore-diameter beads, (**B**) 12-nm pore-diameter beads, and (**C**) 30-nm pore-diameter beads as base materials.

To further investigate the elution profiles of analytes from columns packed with beads of various pore diameters, the elution of benzodiazepines from the column was observed (Figs. 4 and 5, Supplementary Fig. S5). Because benzodiazepines have different basic chemical structure and hydrophobicity from that of steroids (Supplementary Table S3), benzodiazepines have possibility to exhibit different elution behavior from steroids on various types of pore dimeter beads at various temperature. Consequently, similar tendency of elution behavior and retention time change with column temperature was observed. The retention time of benzodiazepines increased with increasing temperature for the 12- and 30-nm pore-diameter beads and decreased with increasing temperature for the 7-nm pore-diameter beads.

**Figure 6:**
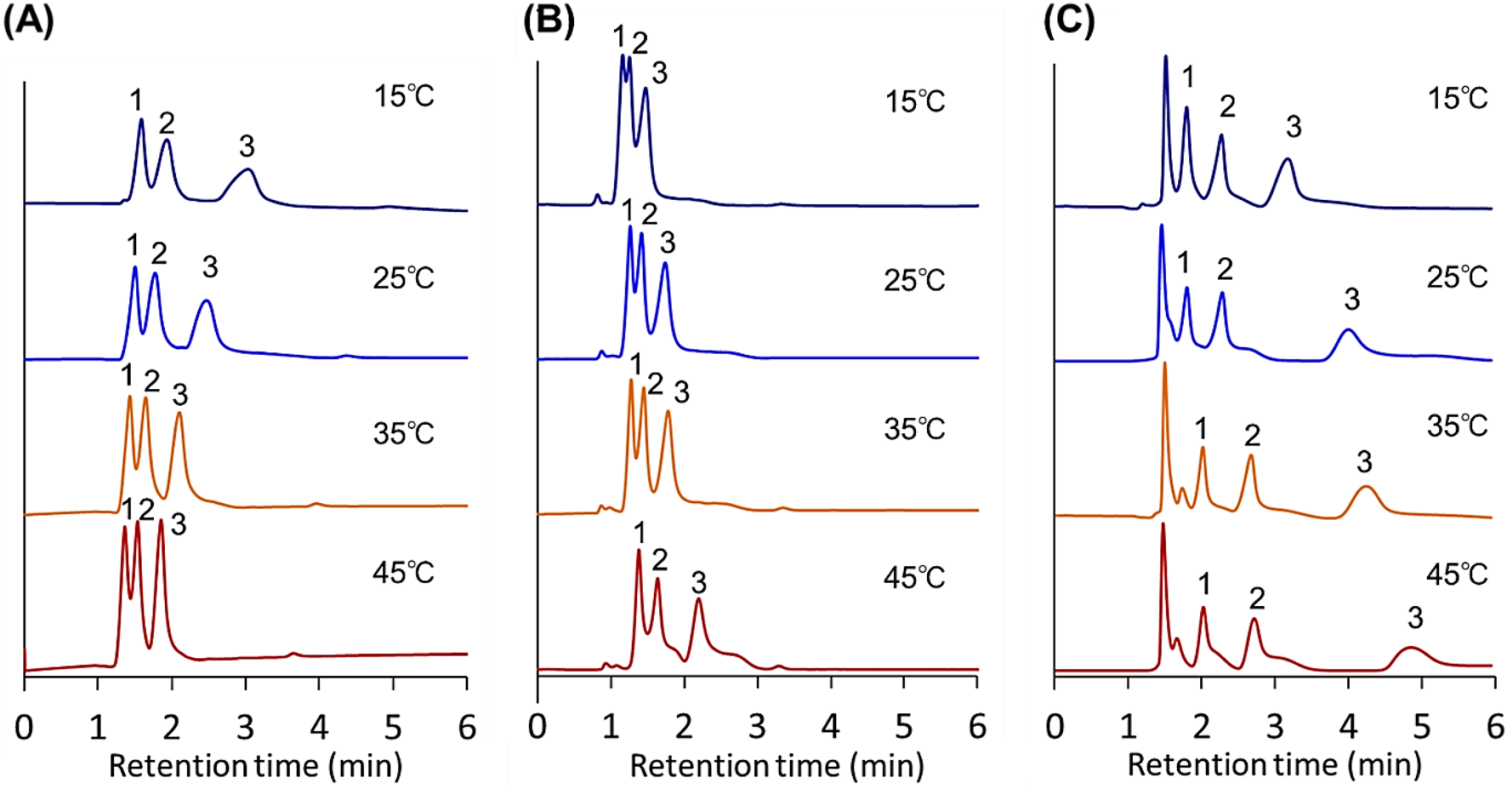
Chromatograms of barbiturates using P(NIPAAm-*co*-BMA) hydrogel-modified bead columns with (**A**) 7-nm pore-diameter beads, (**B**) 12-nm pore-diameter beads, and (**C**) 30-nm pore-diameter beads as base materials. Mobile phase was pure water. Elution of analytes was monitored at 220 nm at a mobile-phase flow rate of 1.0 min/L. Peak 1 = barbital; 2 = allobarbital; and 3 = phenobarbital.

**Figure 7:**
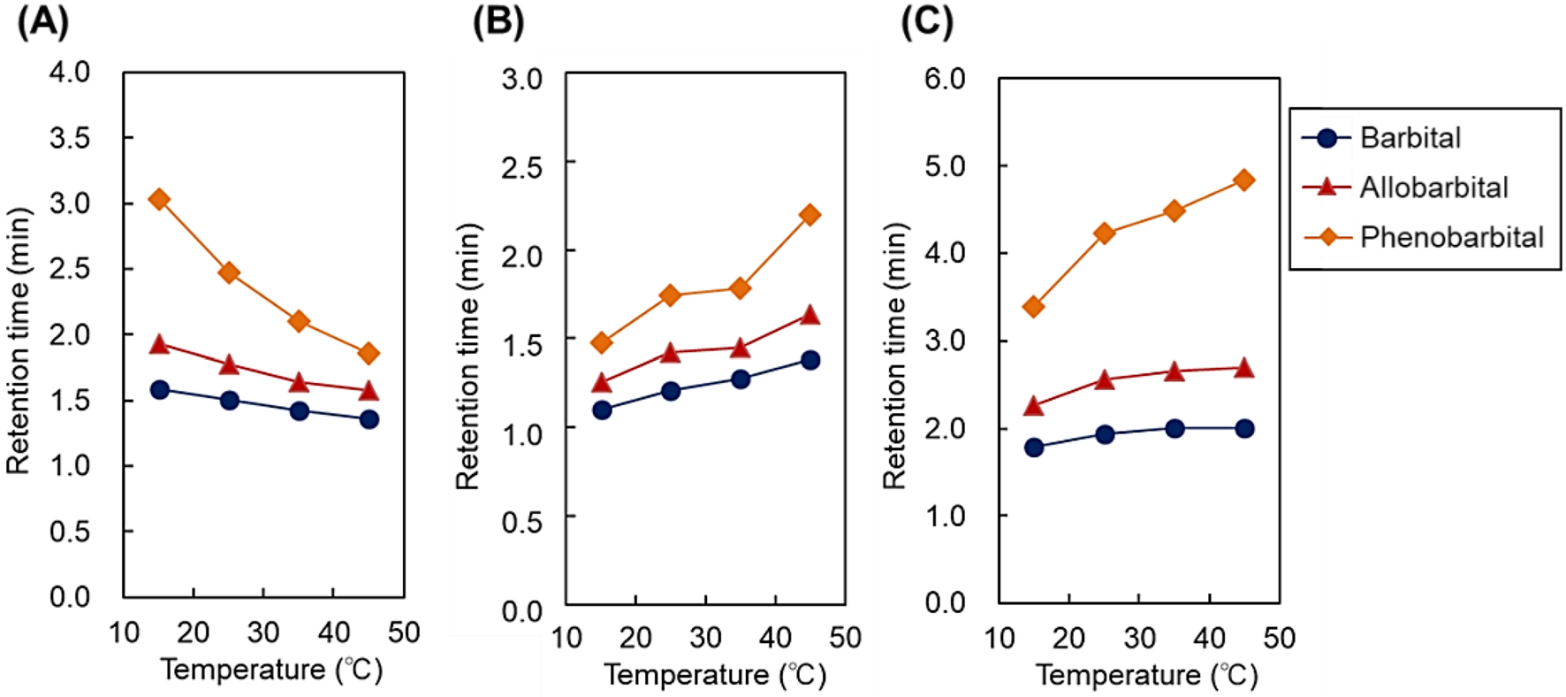
Retention time of barbiturates using P(NIPAAm-*co*-BMA) hydrogel-modified bead columns with (**A**) 7-nm pore-diameter beads, (**B**) 12-nm pore-diameter beads, and (**C**) 30-nm pore-diameter beads as base materials.

Furthermore, the elution behavior of the barbiturates was observed as additional analytes (Figs. 6 and 7, Supplementary Fig. S6) because barbiturates have a different basic chemical structure and smaller hydrophobicity compared to steroids and benzodiazepines (Table S4). For the 12-nm pore-diameter beads, three barbiturates were eluted as an overlapped peak at 15 °C. However, with increasing temperature, the three barbiturate peaks were separated. In contrast, for the 7-nm pore-diameter beads, the separation of the three barbiturates was observed at 15 °C, whereas the peaks moved toward each other and overlapped at high temperatures. Thus, barbiturate separation was performed by modulating the appropriate column temperature for each to the bead pore diameters.

**Figure 8:**
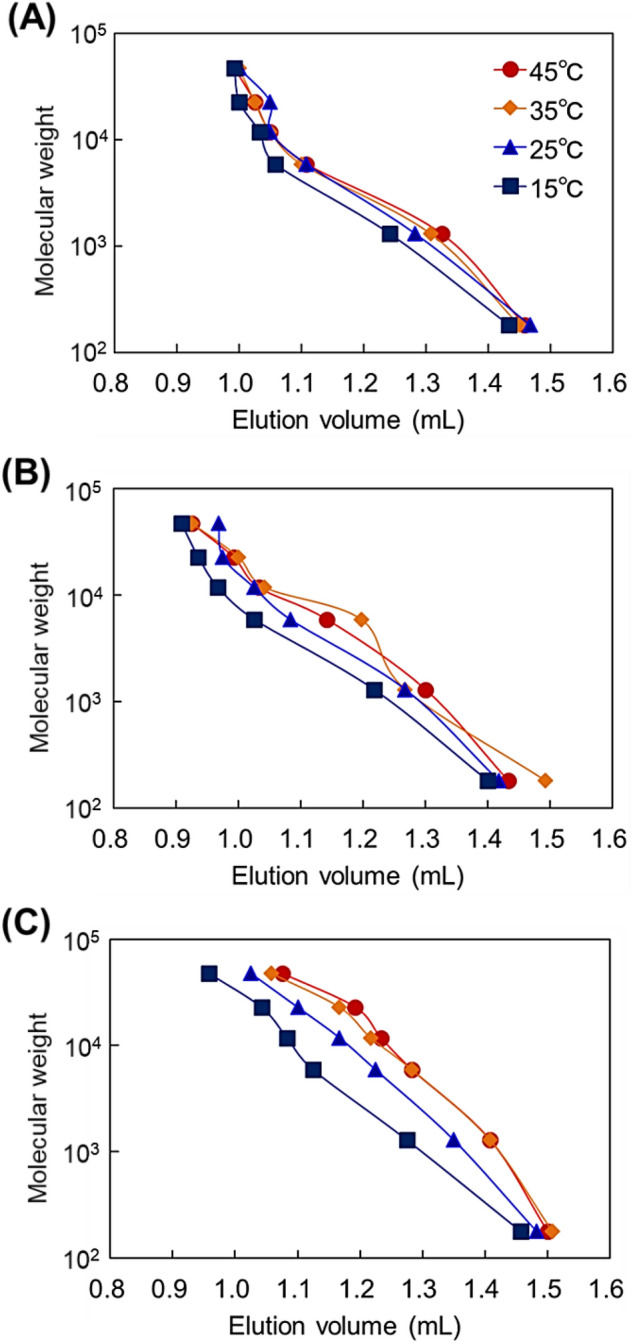
Plots of molecular weight of glucose and pullulan standards and their elution volume through P(NIPAAm-*co*-BMA) hydrogel-modified bead columns with (**A**) 7-nm pore-diameter beads, (**B**) 12-nm pore-diameter beads, and (**C**) 30-nm pore-diameter beads as base materials.

To investigate the diffusion of an analyte into the bead pores, the temperature-dependent elution behavior of pullulan with a range of molecular weights was determined (Fig. 8). Supplementary Table S5 summarizes the molecular weights of glucose and pullulan. All columns exhibited increases in the elution volume of pullulan with increasing column temperature. This is because the modified P(NIPAAm-*co*-BMA) hydrogel inside the pores shrunk, leading to pore size enlargement in the beads and enhanced diffusion of the analyte into the pores. An even larger increase in elution volume with temperature was observed for the columns using large pore-diameter beads. This is because the analyte was more likely to diffuse into large pores than into small pores at high temperatures. Elemental analysis of copolymer hydrogel-modified beads revealed that a similar amount of copolymer was present on the bead surfaces (Table 1). Additionally, the decrease in pore diameter with copolymer hydrogel modification was almost the same among all bead pore sizes (Table 2). These results indicate that the same thickness of copolymer hydrogel was present inside the pores of all three types of beads. In all beads, the copolymer hydrogel inside the pores was shrunk at high temperatures, and analyte diffusion into the pores was thus enhanced compared with the diffusion at low temperatures. However, in the case of the small pore-diameter (7 nm) beads, the pore diameter was not sufficient for analyte diffusion even when the copolymer hydrogel shrank; thus, the small pore-diameter beads did not exhibit large increases in elution volume with increasing temperature.

To investigate temperature-dependent property changes in the hydrogel-modified beads, the dispersity of the prepared beads in water was determined at 15 °C and 45 °C (Supplementary Fig. S7). At 15 °C, all beads were dispersed in water, which was attributed to the hydration of the copolymer hydrogel on the bead surface. By contrast, at 45 °C, the 7-nm pore-diameter beads exhibited poorer dispersity than did the 12- and 30-nm pore-diameter beads. This is probably because the hydrophobicity inside the pore prevented infiltration of water into small pores, whereas large pores allowed for the greater infiltration of water even under hydrophobic conditions within the pore.

These results indicate that the thermoresponsive copolymer modified silica beads with a small pore diameter (7 nm) exhibited inverse temperature-dependent analyte retention profiles compared with those with relatively large pore diameters (12 and 30 nm). The difference in elution profiles was attributed to analyte diffusion into the pores.

The changes in retention profiles during temperature-responsive chromatography were affected by three factors, i.e., (1) hydrophobic interaction, (2) analyte diffusion into pores, and (3) analyte solubility in the mobile phase. (1) The enhancement of the hydrophobic interaction between the polymer and analyte was attributed to the dehydration of the polymer with increasing temperature, which led to an increase in retention time with temperature. (2) Additionally, analyte diffusion into the pores was enhanced with increasing temperature, leading to an enlarged effective area for hydrophobic interaction between the polymer and analyte. (3) By contrast, analyte solubility in the mobile phase increased with increasing temperature, leading to a decrease in analyte retention. Analyte retention during temperature-responsive chromatography resulted from a combination of these factors. In the case of the 12- and 30-nm pore-diameter beads, these three factors affected analyte retention. With increasing temperature, increased hydrophobic interaction between the copolymer and analyte was attributed to the dehydration of the copolymer and an increase in the effective surface area, leading to an increase in analyte retention. By contrast, for the 7-nm pore-diameter beads, the diffusion of the analyte into the pores was relatively suppressed compared with that for the larger-pore-diameter beads. Hence, with the 7-nm pore-diameter beads, the effective surface area for hydrophobic interaction did not increase with increasing temperature. By contrast, analyte solubility into the mobile phase increased with increasing temperature, leading to a decreased retention of the analyte.

The results indicate that bead pore diameter influences the retention behavior of analytes in temperature-responsive chromatography. Using relatively large pore diameter (12 and 30 nm) beads as packing materials, the retention time of an analyte can increase with increasing temperature. By contrast, when using small pore diameter (7 nm) beads, retention time can decrease with increasing temperature, which has not been observed in temperature-responsive chromatography. The small pore-diameter-bead packed column achieved high-resolution separation at low temperatures, whereas the relatively large pore-diameter-bead packed columns (12 and 30 nm) exhibited low-resolution separation at high temperatures. Thus, the development of small pore-diameter-bead packed columns would be useful for the separation of analytes at low temperatures, which has advantages for the separation of analytes that tend to deactivate and decompose at relatively high temperatures.

## Conclusions

The effect of pore diameter of base silica beads on analyte retention in temperature-responsive chromatography was investigated using silica beads with pore sizes of 7, 12, and 30 nm. Elemental analysis indicated that almost the same amount of copolymer hydrogel was applied to the beads independent of bead pore size. Nitrogen adsorption analysis revealed that the pores of the silica beads after hydrogel modification did not become clogged, although pore diameter slightly decreased. The elution behavior of analytes from bead-packed columns was observed using steroids, benzodiazepines, and barbiturates as analytes. Differences in temperature-dependent elution profiles were observed between the column packed with 7-nm pore-diameter beads and those packed with 12- and 30-nm pore-diameter beads. For the columns packed with 12- and 30-nm beads, the retention time of the analytes increased with increasing temperature, whereas retention time decreased with increasing temperature for the columns packed with 7-nm pore-diameter beads. To investigate analyte diffusion into the bead pores, the temperature-dependent elution volume of pullulan was observed. The elution volume of pullulan indicated that its diffusion into pores was enhanced with increasing temperature for the 12- and 30-nm pore-diameter beads owing to the shrinkage of the polymer hydrogel inside the pores. By contrast, for the 7-nm pore-diameter beads, the enhancement of analyte diffusion into the pores was relatively small compared with that for the 12- and 30-nm pore-diameter beads. These results suggest that small pore-diameter beads can obtain the high-resolution separation of analytes at low temperatures, whereas large pore-diameter beads can separate analytes at high temperatures. Thus, the development of small pore-diameter beads would be useful for the separation of analytes at low temperatures, which has advantages for the separation of analytes that tend to deactivate and decompose at relatively high temperatures.

## Methods

### Preparation of thermoresponsive hydrogel-modified beads

P(NIPAAm-*co*-BMA) hydrogel-modified silica beads with various pore diameters were prepared by the following procedure (Fig. 1). The Supplementary Information summarizes the details of all materials. 4,4′-Azobis(4-cyanovaleric acid) (V-501) (3.50 g, 12.5 mmol) and 2-ethoxy-1-ethoxycarbonyl-1,2-dihydroquinoline (6.18 g, 25.0 mmol) were dissolved in 50 mL of *N*,*N*-dimethylformamide in a 200 mL flask. Amino propyl silica beads (5.0 g) were dispersed in the solution. The bead dispersion was degassed by N_2_ gas bubbling in an ultrasonic bath for 30 min. Then, the flask was sealed, and the V-501 immobilization reaction was proceeded with continuous shaking for 17 h at 25 °C. After the reaction, V-501 modified beads were filtered and rinsed with ethanol. The beads were dried at 25 °C in vacuo.

NIPAAm (3.75 g, 33.1 mmol), *n*-butyl methacrylate (0.31 g, 2.18 mmol), and *N,N′*-methylenebisacrylamide (0.135 g, 0.876 mmol) were dissolved in 50 mL of ethanol in a 100 mL flask. Then, V-501 immobilized silica beads (2.0 g) were dispersed in the reaction solution and the solution was degassed by N_2_ gas bubbling in an ultrasonic bath for 30 min. Next, polymerization was proceeded for 15 h at 70 °C. After polymerization, the beads were filtered and rinsed using methanol. The beads were dried in vacuo, and P(NIPAAm-*co*-BMA) hydrogel-modified beads were obtained.

## Characterization of thermoresponsive hydrogel polymer modified beads

The prepared silica beads were characterized by CHN elemental analysis, FE-SEM observation, and nitrogen adsorption.

The carbon composition of the beads was obtained via CHN elemental analysis (PE-2400, PerkinElmer, Waltham, MA, USA). The amount of immobilized V-501 on the silica beads was obtained using the following equation:

1 $${\text{Amount}}\;{\text{ of }}\;{\text{initiator }} = \frac{{\% C_{I} }}{{\% C_{I} (calcd) \times \left( {1 - \% C_{I} /\% C_{I} (calcd)} \right) \times S}}$$

where %*C*_*I*_ is the increase in carbon composition after V-501 immobilization reaction, %*C*_*I*_ (*calcd*) is the calculated carbon percentage of V-501, and *S* is the surface area of the beads.

The amount of modified thermoresponsive hydrogel was obtained as follows:

2 $${\text{Amount }}\;{\text{of }}\;\;{\text{polymer }} = \frac{{\% C_{P} }}{{\% C_{P} (calcd) \times \left( {1 - \% C_{P} /\% C_{P} (calcd) - \% C_{I} /\% C_{I} (calcd)} \right) \times S}}$$

where %*C*_*p*_ is the increase in the carbon composition of the copolymer hydrogel-modified beads relative to that of the V-501 modified beads and %*C*_*P*_ (*calcd*) is the calculated percentage of carbon in the polymer hydrogel.

The bead morphologies of aminopropyl group modified beads and polymer hydrogel-modified beads were observed via FE-SEM (JEOL JSM-7500F, JEOL, Tokyo, Japan).

Pore diameter, pore volume, and surface area were measured by nitrogen adsorption (Belsorp-maxII, MicrotacBEL, Osaka, Japan). These parameters were calculated using the BET method^41,42^.

### HPLC analysis using prepared bead-packed columns

Prepared polymer hydrogel-modified beads were packed into stainless-steel columns (4.6 mm diameter × 100 mm length). Beads were suspended in a methanol/water (1:1) solution. Then, the suspension was poured into a column packer connected to the column. Bead packing was performed by flowing the methanol/water solution at a constant pressure of 350 kg/cm for 1 h. The bead-packed column was connected to an HPLC system (Prominence LC-2030C, Shimadzu, Kyoto, Japan). The elution behavior of analytes from the column was observed using steroids (Table S1), benzodiazepines (Table S3), and barbiturates (Table S4). Pure water was used as the mobile phase at a flow rate of 1.0 mL/min. The elution behavior of the steroids, benzodiazepines, and barbiturates was detected at 254, 220, and 220 nm, respectively.

To investigate analyte retention behavior on the column, Van’t Hoff plots for the analytes were obtained. The retention factor *k’* was obtained using the following equation:

3 $${\text{Retention }}\;{\text{factor }} = \frac{{t_{R} - t_{0} }}{{t_{0} }}$$

where *t*_*R*_ is the analyte retention time and *t*_*0*_ is the retention time of uracil as an initial standard^45^.

Additionally, the elution of glucose and pullulan standard samples from the columns was observed to investigate the molecular weight of the analytes and the pore size of the beads. Pullulan was detected using an RI detector (830-RI, Jasco, Tokyo, Japan).

## Supplementary Information

Supplementary Information.
