# Supplementary material for: Effect of pore diameter on the elution behavior of analytes from thermoresponsive polymer grafted beads packed columns
Source: Sci Rep. 2021 May 11;11:9976. doi: 10.1038/s41598-021-89165-9 (PMC8113370; doi:10.1038/s41598-021-89165-9)
Supplement: Supplementary file 1 — Supplementary Information. [file 41598_2021_89165_MOESM1_ESM.docx]

Supplementary Information

**Effect of pore diameter on the elution behavior of analytes from thermoresponsive polymer grafted**

**beads packed columns**

Kenichi Nagase*, Yuta Umemoto, and Hideko Kanazawa

Faculty of Pharmacy, Keio University, 1-5-30 Shibakoen, Minato, Tokyo 105-8512, Japan.

*Corresponding author: (Phone) +81-3-5400-1378; (E-mail) nagase-kn@pha.keio.ac.jp

**Materials**

*N*-Isopropylacrylamide (NIPAAm) was kindly provided by KJ Chemicals (Tokyo, Japan) and was purified by recrystallization from *n*-hexane. *n*-Butyl methacrylate, *N,N’*-methylenebisacrylamide, 4,4’-azobis(4-cyanovaleric acid) (V-501), uracil, steroids, and benzodiazepines were obtained from Fujifilm Wako Pure Chemicals (Osaka, Japan). *n*-Hexane, *N*,*N*-dimethylformamide, and ethanol were obtained from Kanto Chemicals (Tokyo, Japan). 2-Ethoxy-1-ethoxycarbonyl-1,2-dihydroquinoline was obtained from Sigma Aldrich (St Louise, MO, USA). Aminopropyl group modified silica beads (diameter: 5 μm; pore size: 70, 120, and 300 Å) and stainless-steel columns (inner diameter: 4.6 mm; column length: 100 mm) were purchased from Nishio Kogyo (Tokyo, Japan).


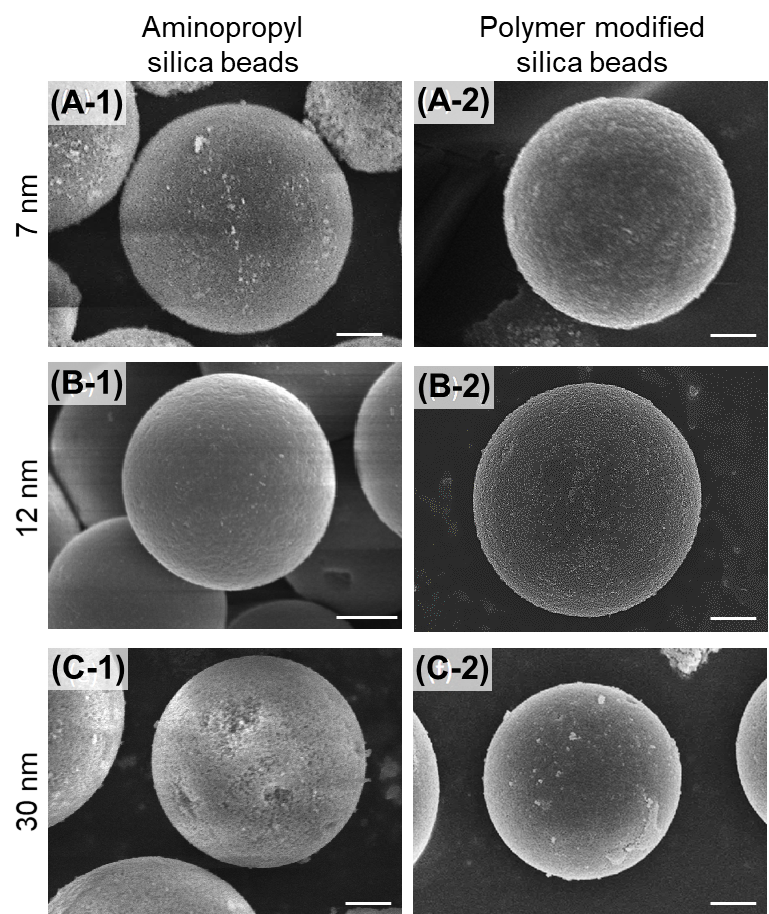


**Supplementary Figure S1.** Field emission scanning electron microscopy images of (A) 7-nm pore-diameter beads, (B) 12-nm pore-diameter beads, and (C) 30-nm pore-diameter beads. (-1) Unmodified amino propyl silica beads and (-2) copolymer hydrogel-modified beads.

**Supplementary Table S1.** Properties of hydrophobic steroids

| Compounds | Structure | Molecular weight | Log*P* ^a^ |
| --- | --- | --- | --- |
| Hydrocortisone | 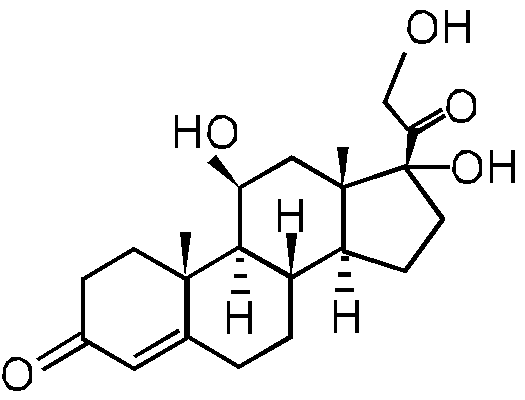 | 362.46 | 1.61 |
| Prednisolone | 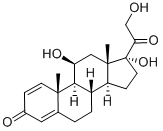 | 360.44 | 1.62 |
| Dexamethasone | 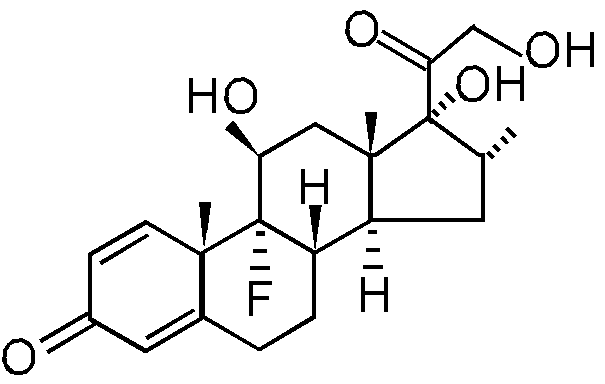 | 392.46 | 1.83 |
| Hydrocortisone acetate | 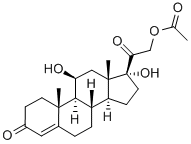 | 404.50 | 2.30 |
| Testosterone | 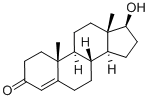 | 288.42 | 3.32 |

a) Partition coefficient in an *n*-octanol/water system.

**Supplementary Table S2.** Retention time of steroids with repeated measurement.

| Analyte | 10°C | | 40°C | |
| --- | --- | --- | --- | --- |
|  | Retention time  (min) ^a)^ | RSD  (%) | Retention time  (min) ^a)^ | RSD  (%) |
| Hydrocortisone | 2.842 | 0.227 | 3.928 | 0.427 |
|  | 2.830 |  | 3.936 |  |
|  | 2.828 |  | 3.951 |  |
|  | 2.828 |  | 3.960 |  |
|  | 2.826 |  | 3.969 |  |
| Prednisolone | 3.467 | 0.055 | 4.916 | 0.348 |
|  | 3.469 |  | 4.957 |  |
|  | 3.472 |  | 4.951 |  |
|  | 3.470 |  | 4.939 |  |
|  | 3.468 |  | 4.925 |  |
| Dexamethasone | 5.559 | 0.599 | 8.012 | 0.404 |
|  | 5.483 |  | 7.940 |  |
|  | 5.488 |  | 7.941 |  |
|  | 5.481 |  | 7.938 |  |
|  | 5.492 |  | 7.942 |  |
| Hydrocortisone acetate | 6.330 | 0.859 | 10.654 | 0.937 |
|  | 6.344 |  | 10.422 |  |
|  | 6.329 |  | 10.513 |  |
|  | 6.429 |  | 10.580 |  |
|  | 6.438 |  | 10.654 |  |
| Testosterone | 12.890 | 0.129 | 21.408 | 0.169 |
|  | 12.898 |  | 21.484 |  |
|  | 12.895 |  | 21.451 |  |
|  | 12.916 |  | 21.419 |  |
|  | 12.930 |  | 21.394 |  |

a) Measured using a P(NIPAAm-co-BMA) hydrogel-modified bead-packed column with a 4.6-mm inner diameter and 50-mm column length. The mobile phase was pure water with a flow rate of 1.0 mL/min, and the detection was 254 nm.


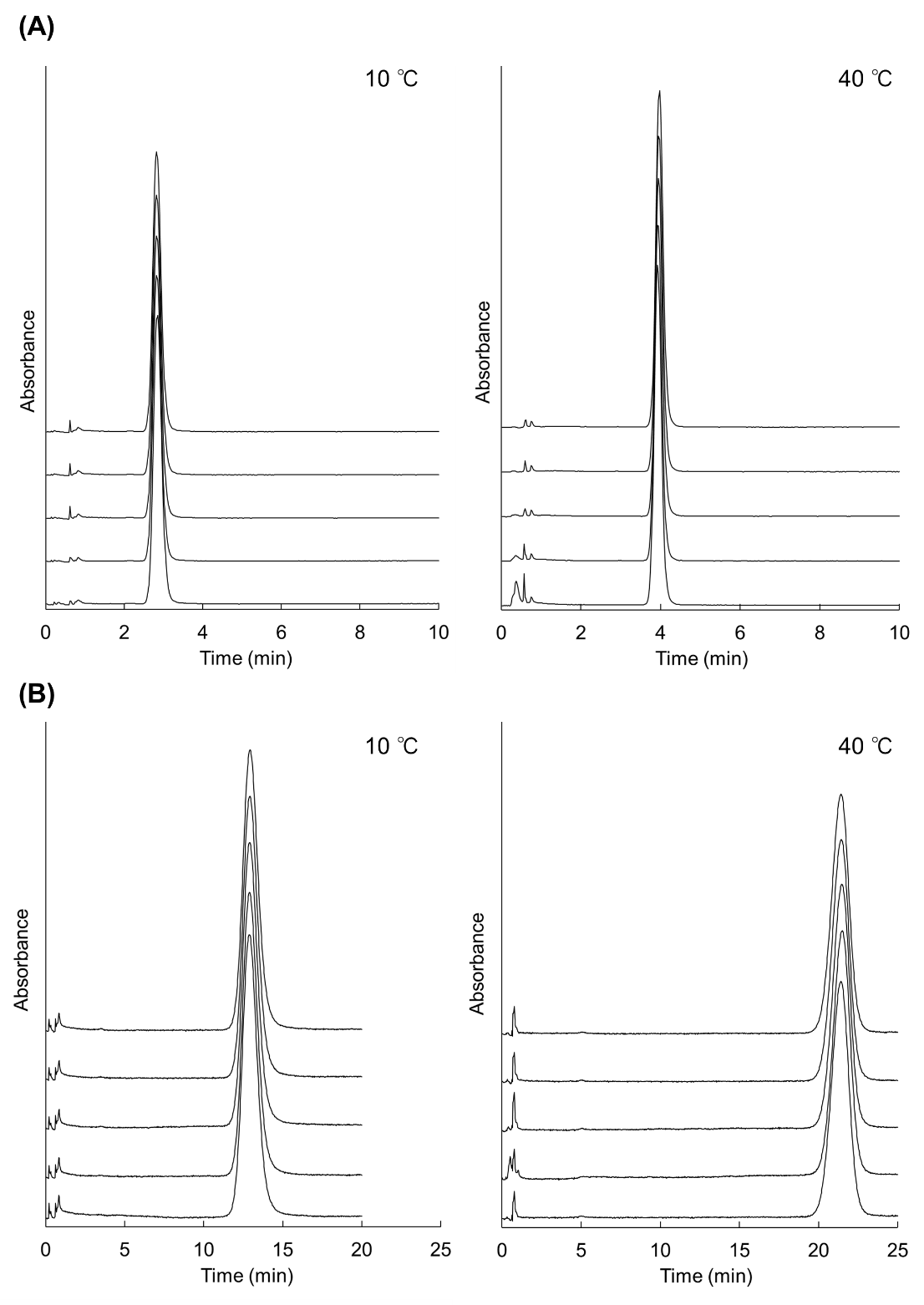


**Supplementary Figure S2.** Chromatogram of steroids with repeated measurement on P(NIPAAm-*co*-BMA) hydrogel-modified bead-packed column with a 4.6-mm inner diameter and 50-mm column length. (A) hydrocortisone and (B) testosterone. The mobile phase was pure water with a flow rate of 1.0 mL/min, and the detection was 254 nm.


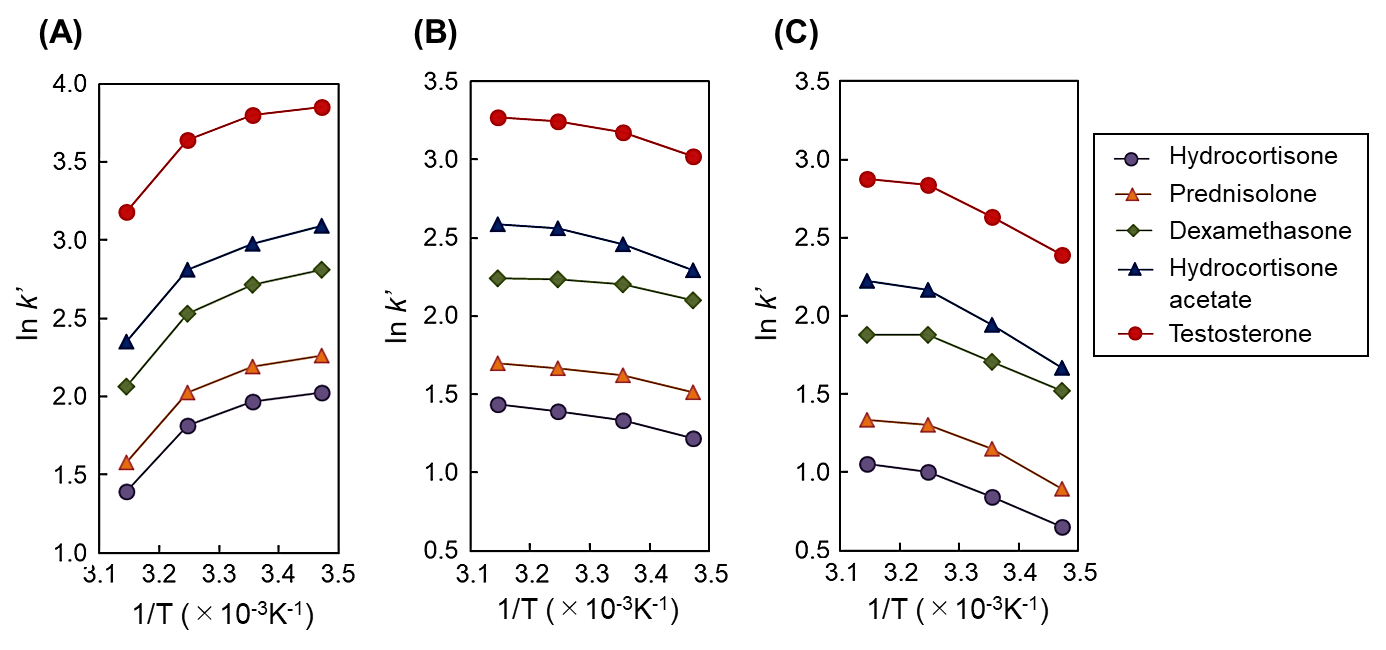


**Supplementary Figure S3.** Van’t Hoff plots of steroids on P(NIPAAm-*co*-BMA) hydrogel-modified bead columns. (A) 7-nm pore-diameter beads, (B) 12-nm pore-diameter beads, and (C) 30-nm pore-diameter beads as base materials.


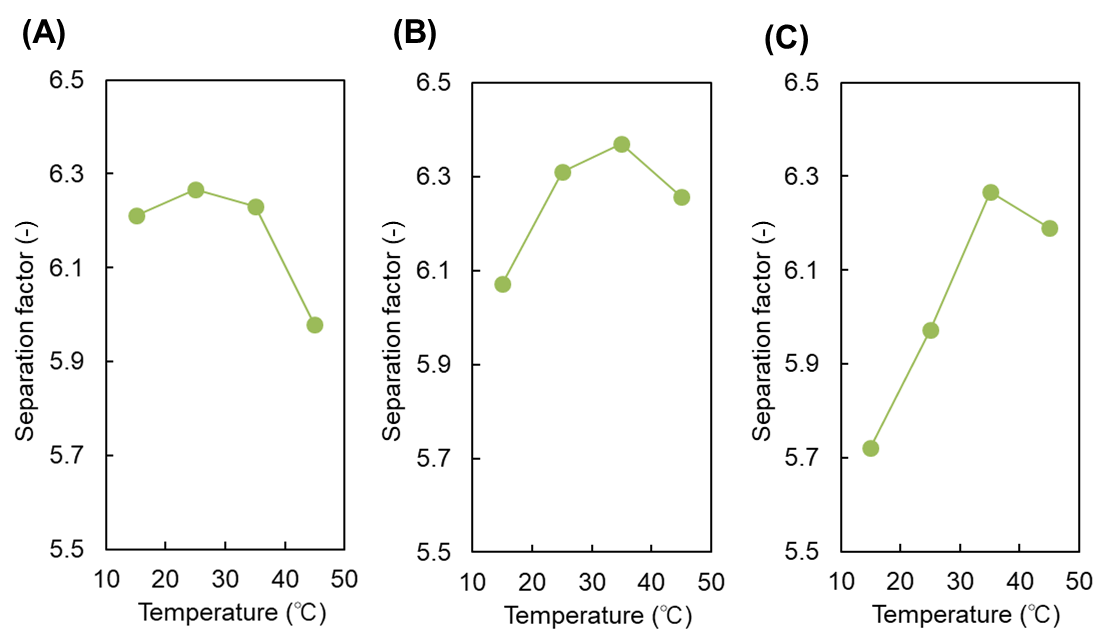


**Supplementary Figure S4.** Separation factor between hydrocortisone and testosterone on P(NIPAAm-*co*-BMA) hydrogel-modified bead columns. (A) 7-nm pore-diameter beads, (B) 12-nm pore-diameter beads, and (C) 30-nm pore-diameter beads as base materials.

**Supplementary Table S3.** Properties of benzodiazepines

| Compounds | Structure | Molecular weight | Log*P* ^a^ |
| --- | --- | --- | --- |
| Flunitrazepam | 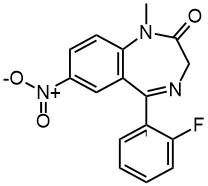 | 313.28 | 2.31 |
| Diazepam | 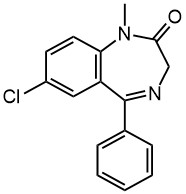 | 284.74 | 2.77 |

a) Partition coefficient in an *n*-octanol/water system.


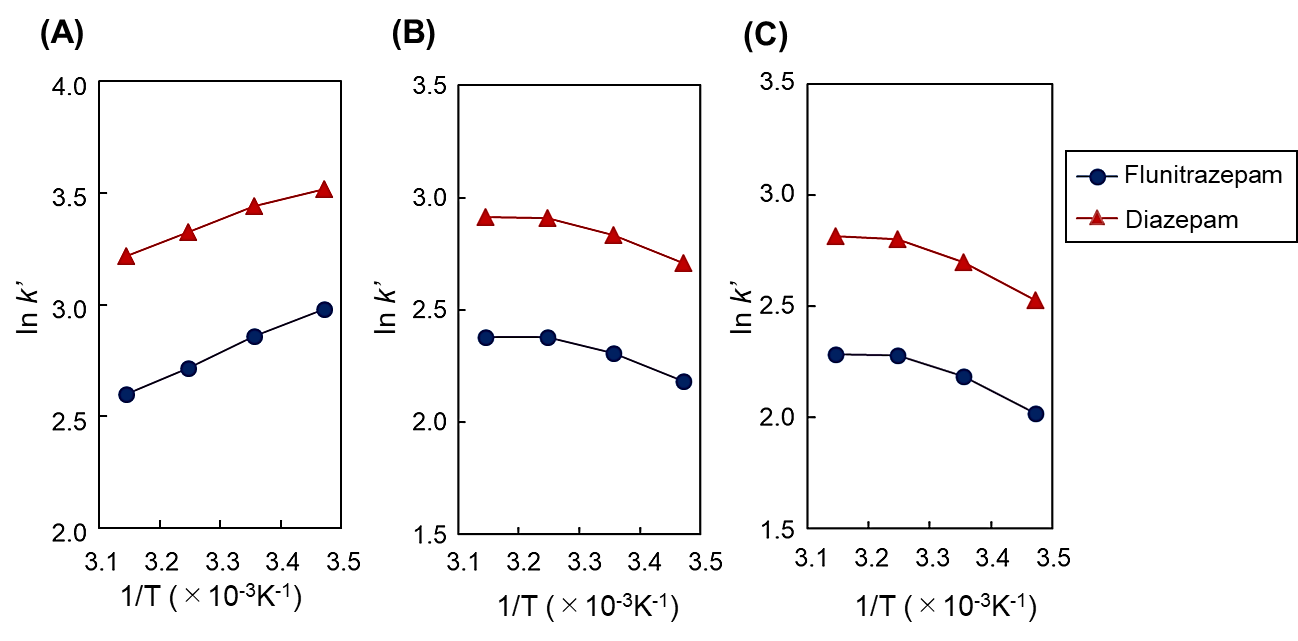


**Supplementary Figure S5.** Van’t Hoff plots of benzodiazepines on P(NIPAAm-*co*-BMA) hydrogel-modified bead columns. (A) 7-nm pore-diameter beads, (B) 12-nm pore-diameter beads, and (C) 30-nm pore-diameter beads as base materials.

**Supplementary Table S4.** Barbiturate properties

| Compounds | Structure | Molecular weight | Log*P* ^a^ |
| --- | --- | --- | --- |
| Balbital | 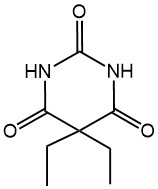 | 184.20 | 0.75 |
| Allobarbital | 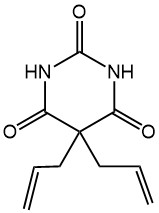 | 208.22 | 1.04 |
| Phenobarbital | 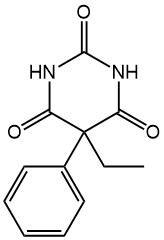 | 232.24 | 1.52 |

^a^ partition coefficient in an *n*-octanol/water system


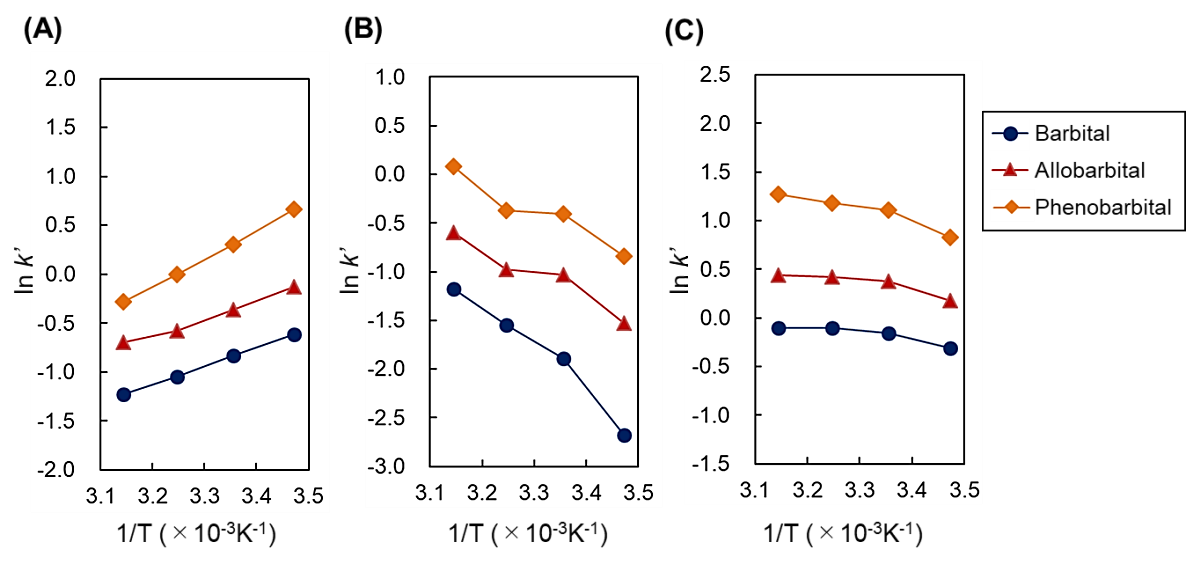


**Supplementary Figure S6.** Van’t Hoff plots of barbiturates on P(NIPAAm-*co*-BMA) hydrogel-modified bead columns. (A) 7-nm pore-diameter beads, (B) 12-nm pore-diameter beads, and (C) 30-nm pore-diameter beads as base materials.

**Supplementary Table S5.** Molecular weight and radius of gyration (Rg) of glucose and pullulan samples

| Analyte | Molecular weight ^a)^ | Rg (nm)^b)^ |
| --- | --- | --- |
| Glucose | 180 | 0.3 |
| P-1^c)^ | 1300 | 0.9 |
| P-5 | 5900 | 2.3 |
| P-10 | 11800 | 3.4 |
| P-20 | 22800 | 5.0 |
| P-50 | 47300 | 7.6 |

a) Manufacture’s data. b) Calculated using the relation between Mw and Rg of pullulan C) P-1 to P-50 indicate pullulan molecular weight.


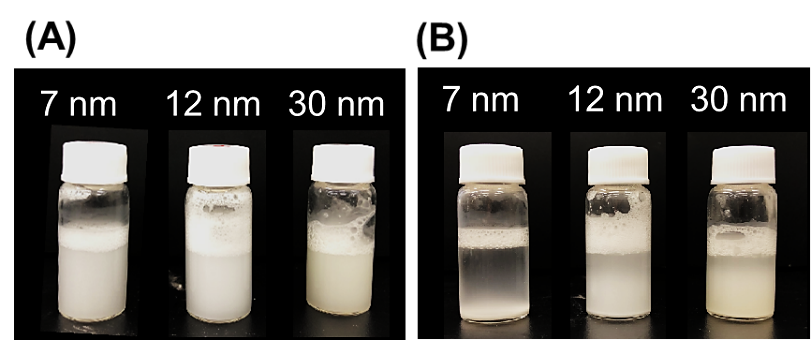


**Supplementary Figure S7.** Dispersion of the beads at (A) 15°C and (B) 45°C.
